# Caregivers' practices, knowledge and beliefs of antibiotics in paediatric upper respiratorytract infections in Trinidad and Tobago: a cross-sectional study

**DOI:** 10.1186/1471-2296-5-28

**Published:** 2004-12-01

**Authors:** Neeta Parimi, Lexley M Pinto Pereira, P Prabhakar

**Affiliations:** 1 Bishop Anstey High School, Port of Spain, Trinidad; 2 Department of Paraclinical Sciences, Faculty of Medical Sciences, The University of the West Indies, St Augustine, Trinidad; 3 Caribbean Epidemiology Center (PAHO/ WHO), Port of Spain, Trinidad; 4 RNA Center, Case Western Reserve University, Cleveland, OHIO, USA

## Abstract

**Background:**

Antibiotic overuse and misuse for upper respiratory tract infections in children is widespread and fuelled by public attitudes and expectations. This study assessed knowledge, beliefs, and practices regarding antibiotic use for these paediatric infections among children's caregivers' in Trinidad and Tobago in the English speaking Caribbean.

**Methods:**

In a cross-sectional observational study, by random survey children's adult caregivers gave a telephone interview from November 1998 to January 1999. On a pilot-tested evaluation instrument, respondents provided information about their knowledge and beliefs of antibiotics, and their use of these agents to treat recent episodes (< previous 30 days) of upper respiratory tract infections in children under their care. Caregivers were scored on an antibiotic knowledge test and divided based on their score. Differences between those with high and low scores were compared using the chi-square test.

**Results:**

Of the 417 caregivers, 70% were female and between 18–40 years, 77% were educated to high school and beyond and 43% lived in urban areas. Two hundred and forty nine (60%) respondents scored high (≥ 12) on antibiotic knowledge and 149 (34%) had used antibiotics in the preceding year. More caregivers with a high knowledge score had private health insurance (33%), (p < 0.02), high school education (57%) (p < 0.002), and had used antibiotics in the preceding year (p < 0.008) and within the last 30 days (p < 0.05). Caregivers with high scores were less likely to demand antibiotics (p < 0.05) or keep them at home (p < 0.001), but more likely to self-treat with antibiotics (p < 0.001). Caregivers administered antibiotics in 241/288 (84%) self-assessed severe episodes of infection (p < 0.001) and in 59/126 (43%) cough and cold episodes without visiting a health clinic or private physician (p < 0.05).

**Conclusions:**

In Trinidad and Tobago, caregivers scoring low on antibiotic knowledge have erroneous beliefs and use antibiotics inappropriately. Children in their care receive antibiotics for upper respiratory tract infections without visiting a health clinic or a physician. Educational interventions in the community on the consequences of inappropriate antibiotic use in children are recommended. Our findings emphasise the need to address information, training, legislation and education at all levels of the drug delivery system towards discouraging self-medication with antibiotics in children.

## Background

Acute respiratory infections and diarrhoeal diseases are the leading causes of childhood mortality, resulting in 25–33% of all deaths in children in developing countries. Worldwide, antibiotics are the most commonly prescribed and abused drugs for upper respiratory tract infections (URTIs) [1,2]. In surveys on antibiotic use, about 20 % of prescriptions were inappropriate [3] and a high proportion of patients received antibiotics during clinic visits [4].

A significant force driving the occurrence and the spread of antibiotic resistance is the inappropriate use of antibiotics in primary and ambulatory care settings. *Streptococcus pneumoniae*, *Hemophilus influenzae *and *Moraxella catarrhalis*, the most common bacterial pathogens causing acute bacterial rhinosinusitis (ABRS) in children, in both the developed and developing countries, have already demonstrated resistance to the first line antibiotics [5-9]. The Sinus and Allergy Health Partnership (SAHP) guidelines for the treatment of ABRS observe antibiotics prescribed for ABRS are not only ineffective, but may contribute to the development of antibiotic-resistant bacterial infections [10] which is supported by the increasing resistance of *Streptococcus pneumoniae *and the increasing prevalence of strains resistant to the beta-lactams and co-trimoxazole. Unnecessary antibiotic use in viral respiratory illnesses in humans is a key factor influencing the emergence and spread of resistant pneumococci. Inappropriate antibiotic use may be consequent to misdiagnosis of the illness (viral and bacterial URTIs present with similar symptoms), patients' expectations, and their demands which induce physicians to prescribe antibiotics [11].

In the United States, a higher proportion of infections due to penicillin resistant pnuemococci among young children and whites have been attributed to an overuse of antibiotics [12,13]. Epidemiological studies demonstrate recent antibiotic use is strongly associated with carriage of resistant pneumococci in the community and the individual, and in patients with invasive pneumococcal disease, recent antibiotic use has been associated with increased risk of infection with a resistant strain [14].

Previous community-oriented studies suggest an irrational use of antibiotics, particularly in the developing and lesser-developed countries. Factors influencing antibiotic resistance are the higher incidence of infectious diseases in children, the lack of access to health care, costs and poor regulatory controls on the use of prescription drugs such as antibiotics, coupled with low antibiotic knowledge prompting increased self medication with these drugs [15-17]. As the first health-decision makers in deciding when to initiate antibiotic treatment and limit their unnecessary use, mothers and caregivers must have the appropriate knowledge to enable correct decisions. This first Caribbean study investigated the knowledge, beliefs and practices of children's caregivers in Trinidad and Tobago regarding antibiotic utilisation and explored these beliefs in self-administration of antibiotics in childhood URTIs. Gaining a better understanding of caregivers' management of childhood URTIs and factors that influence their use of antibiotics will allow appropriate educational interventions and reduce unnecessary antibiotic use in children.

## Methods

### Setting

Trinidad and Tobago, a twin island republic in the Caribbean located off the Venezuelan coast with a population of 1.3 million people is the second largest country in the English speaking Caribbean. About 74% of the country's population live in the urban areas and 60% of the population is aged between 15–64 years. The literacy rate in Trinidad and Tobago is 98% and at least 70% of the country's adult population has completed secondary (high) school to the pre-university level. Medical care in Trinidad and Tobago is publicly financed through three (3) regional health authorities (North West, South West and Eastern regions) in Trinidad, and one in Tobago. Secondary and tertiary care are provided at one general hospital in Port-of-Spain, one in San Fernando (1,245 beds), and at two county hospitals in Trinidad (111 beds), and at one hospital in Tobago (96 beds), besides institutions for specialised services. Primary health care is provided at 82 health centers in Trinidad and 19 in Tobago. The ratio of the population to a health center ranges from < 3000 per center in Tobago to > 21,000 per center in the north-west region. There are 33 private hospitals and approximately 45% of the population preferentially uses the private sector services. Private general practitioners are concentrated in the cities and larger towns and the estimated ratio of physician per population is 7.5 per 10, 000 inhabitants [18].

### Design

This prospective cross sectional observational study was conducted in Trinidad and Tobago from November 1998 to January 1999 in randomly selected subjects interviewed over the telephone. The study design and methods have been described previously [19]. Briefly, a sample size of 800 participants with a working telephone was calculated based on 80% power to detect a difference of at least 3% use of antibiotics giving an error of 0.05. This being the first such telephone survey in the country, with no experience of the rejection rate, 1,600 telephone numbers were randomly obtained from a sampling frame of 167,272 telephone subscribers of The Telecommunication Services of Trinidad and Tobago, the only telephone service provider in the country. Of 824 respondents, 753 agreed to participate with a response rate of 91.4%. At the outset of the study participants were questioned if they were caring for a child ≤ 12 years and the term 'antibiotic' was explained in a simple sentence : *"Antibiotics are drugs that are prescribed for the treatment of diseases caused by germs"*.

The pilot-tested questionnaire consisted of 42 items in three parts, designed to investigate knowledge, beliefs and practices of antibiotics [19]. The first part on the caregiver's demographic data included the employment status, health insurance and educational background. The second part inquired about caregivers' knowledge and beliefs. To determine antibiotic knowledge participants were asked to identify 4 antibiotics from a list of 8 commonly used drugs, and could attain a maximum score of 16. Caregivers' beliefs about antibiotics were determined using the following three questions: 1. Do you think antibiotics can cure all infections? 2. Do you believe antibiotics are free from side-effects? 3. Do you think antibiotics are generally safe? From our earlier report and the pilot project of the current study respondents differentiated their understanding of 'side-effects' and 'safety'. The former was associated with unwanted disturbances from drug therapy on the quality of life and the latter was associated with life-threatening issues like organ toxicity and death. The third part of the questionnaire ascertained caregivers' practices of antibiotic use. They were asked about symptoms which children in their care had in the past 30 days, their assessment of the symptom severity, whether they sought medical assistance and if they administered any antibiotic to the child. Respondents were not asked to name the antibiotic. Information on suspected side-effects or allergic reactions was excluded from the final questionnaire as these responses in the pilot study were uncertain and subject to memory recall.

### Analysis

Four hundred and seventeen of the 753 respondents were adult caregivers with children in the family and their responses were analysed. An antibiotic knowledge score was created based on the caregivers' responses to eight common drugs, which included four antibiotics. A high Antibiotic Knowledge Score (AKS) was defined as that at or above the median score. Data were analysed using SPSS version 11.0 (Chicago), and associations were determined by the Chi square test for antibiotic knowledge and caregiver's education, beliefs and practices and recent and past antibiotic use for URTIs.

## Results

The majority of respondents was female (70%), ≥ 31 years, (72%) and had completed high school (pre-university) education (77%). There was a marginally high (57%) representation from the rural area (Table 1), but residence showed no relation to the determinants of the study. The ratio of respondents with African and Indian heritage bore close similarity to the ethnic profile of the population in Trinidad and Tobago. Seventy one percent of caregivers with children did not have private health insurance. Comparable proportions of caregivers, 35% and 34% respectively reported using antibiotics recently (< 30 days) and in the past (within the last one year). The median antibiotic knowledge score was 12 and was determined by the correct identification of penicillin, tetracycline, 'Augmentin', and 'Bactrim' as antibiotics from a list of 8 common drugs. Two hundred and forty-nine (60%) caregivers scored at or above the median score. The significant predictors of high antibiotic knowledge in caregivers were those who were employed, had private health insurance and, high school education. More caregivers with a high AKS had used antibiotics recently (p < 0.037) and in the past (p < 0.008) (Table 2).

**Table 1:** Characteristics of children's caregivers in Trinidad and Tobago.

**Characteristics**	**Caregivers with children (n = 417)**
Number of people in the house (mean +/- SD)	4.89 +/- 1.9
Number of children (mean +/- SD)	2.26 +/- 1.38
**Gender:**	
Males	126 (30)%
Females	291 (70%)*
**Age group**	
18–30 yrs	112 (28%)
31–40 yrs	165 (42%)
> 41 yrs	129 (30%)
**Residence**	
Rural	235 (57%)
Urban	176 (43%)
**Ethnicity**	
African	142 (35%)
Indian	158 (39%)
Others	110 (29%)
**Education**	
Primary school	96 (23%)
High school	228 (55%)*
College	90 (22%)
**Health Insurance**	
With Private Health insurance	114 (29%)
Without Private Health insurance	281 (71%)*
Antibiotics used in previous year	143 (34%)
Antibiotics used in last 30 days	149 (35%)

* Significant at p < 0.05

**Table 2:** Caregivers' Antibiotic Knowledge Score (AKS) and associated factors

**Factors**	**AKS <12(n = 168) (%)**	**AKS ≥ 12(n = 249) (%)**	**p VALUE**
**Gender**			
Males	51 (30)	51 (30)	0.96
Females	117 (70)	117 (70)	
**Residence**			
Rural	75 (46)	101 (41)	0.29
Urban	88 (54)	147 (59)	
**Health Insurance**			
Has insurance	35 (22)	79 (33)	0.02*
Does not have	122 (78)	159 (67)	
**Age Groups**			
18–30 yrs	55 (34)	57 (23)	0.07
31–40 yrs	60 (37)	105 (43)	
>41 yrs	47 (29)	82 (34)	
**Ethnicity**			
African	57 (35)	85 (35)	0.70
Asian	67 (41)	91(37)	
Others	41 (24)	69 (28)	
**Education**			
Primary	53 (37)	43 (17)	0.002*
High School	87 (52)	141 (57)	
Tertiary Education	28 (18)	62 (26)	
**Employment**			
Employed	69 (43)	130 (54)	0.09
Self Employed	28 (18)	40 (17)	
Housewife/retired/unemployed	62 (39)	71 (29)	
**Recent and past antibiotic use**			
Used in the last 12 months	45 (27)	98 (40)	0.008*
Not used in the last 12 months	118 (70)	150 (60)	
Used in the last 30 days	50 (30)	99 (40)	0.037*
Not used in the last 30 days	122 (73)	150 (60)	

Significant at (p < 0.05)

A majority of respondents had correct beliefs regarding whether 'antibiotics cure all infections' (54% [227/417]) and 'antibiotics are free from side effects' (61% [253/417]). Few (11% [49/417]) respondents believed antibiotics are generally safe. and some caregivers (18%–24%) remained non-responsive to all questions. The AKS of caregivers did not influence their beliefs (Table 3). Eighty six caregivers (22%) admitted to demanding antibiotics from a doctor. More caregivers (28%) with a low AKS demanded antibiotic prescriptions (p < 0.05) and kept these drugs at home (33%) (p < 0.001), to treat illnesses. Self-initiation of treatment for URTIs with antibiotics was more frequent (p < 0.05) among caregivers who had a high AKS. Caregiver's knowledge scores were not associated with the use of antibiotics given by relatives and / or friends or with compliance with the course whether recommended by the pharmacist or the doctor. (Table 3).

**Table 3:** Antibiotic beliefs and practices of Caregivers in Trinidad

**FACTORS**	**AKS <12 (%)**	**AKS ≥ 12 (%)**	p value
**1. Antibiotic Beliefs:**			
a. Cure all infections			
Agree	48 (37)	63 (30)	0.17
Disagree	81 (63)	146 (70)	
b. Free from side -effects			
Agree	21 (18)	44 (21)	0.30
Disagree	99 (82)	153 (79)	
c. Generally safe			
Agree	21 (17)	28 (13)	0.40
Disagree	105 (83)	183 (87)	
**2. Antibiotic Practices**			
Demands from doctor for URTI in children			
Yes	41 (28)	45 (19)	0.05*
No	106 (72)	188 (81)	
Keeps at home			
Yes	53 (33)	45 (19)	0.001**
No	107 (67)	195 (91)	
Self treatment			
Yes	33 (12)	69 (32)	
No	154 (88)	150 (68)	0.001**
Given by friends and relatives			
Yes	16 (10)	25 (11)	
No	142 (90)	213 (89)	0.9
Compliance			
Yes	89 (63)	152 (71)	
No	52 (37)	63 (29)	0.13

*Significant at p < 0.05, ** Significant at p < 0.001

Caregivers reported as many as 450 episodes of URTIs in children within the previous 30 days (1.07 episodes per family) and 149 (35%) caregivers self-administered antibiotics in 64% (288/450) of these episodes. Cough and cold was the most frequently reported URTI symptom (48%, 214/450) followed by fever (28%,128/450) and sore throat (24%,108 / 450). Caregivers were more likely to give children antibiotics when they perceived URTIs to be severe [241/288 (84%)] (p < 0.001), and administered these drugs for the common cold [112/136, (82%)] fever [75/87 (83%)] and sore throat [54/65 (86%)] (Table 4). In 16% of episodes which caregivers deemed to be of mild severity, they self-administered antibiotics. Children received antibiotics without visiting a health clinic or a private physician for 126 (44%) URTI episodes, and more frequently for the common cold 59 (43.7%) compared with fever and sore throat (p < 0.05).

**Table 4:** Antibiotic administration by caregivers for severe URTIs (n = 288) and visits to health provider

**URTI Episodes**	**Assessed severe by caregiver (%)**			**Visited a health clinic/private physician (%)**		
	Yes	No	Total	Yes	No	Total
Cough and Cold	112(82)	24(18)	136	77 (57)	59(43)*	136
Fever	75 (83)	12(17)	87	55 (63)	32(37)	87
Sore throat	54 (86)	11(14)	65	30 (46)	35(54)	65
Total	241(84)**	47 (16)	288	162(56)	126(44)	288

* Significant at p < 0.05

** Significant at p < 0.001

## Discussion

This cross sectional study in Trinidad and Tobago determined the antibiotic knowledge of children's caregivers and the influence of this knowledge on their beliefs and use of these agents for URTIs in children under their care. We found high school education and higher socio-economic status (income permitted private health insurance) was significantly associated with higher knowledge scores. Similar associations between knowledge and antibiotic use were reported in a study in the Indian state of Kerala [20]. In the present study more caregivers who scored high on antibiotic knowledge, had used antibiotics (recently and in the past) compared with those who attained a low knowledge score.

A significant proportion of caregivers in the present study had misconceptions that could contribute to the inappropriate use of antibiotics. Equal proportions of caregivers with high and low knowledge scores believed that antibiotics cure all infections and are free from side-effects. Even though URTIs are generally of viral aetiology [21,22], these mistaken beliefs may have steered antibiotic abuse from self treatment or over the counter demands at the pharmacy which are fostered from easy availability of these drugs at community pharmacies in Trinidad and Tobago [19]. In a survey from the United States, 48% of paediatricians reported parents do pressure them to prescribe antibiotics [23], and 78% of the sample believed educating parents on appropriate indications for antibiotic use was the single most important factor to promote suitable prescribing, suggesting effective communication between physicians and parents may reduce inappropriate antibiotic prescribing.

Practices such as demanding a prescription for antibiotics from a physician, and keeping antibiotics at home (hoarding) were higher in caregivers with a low AKS. In Israel Shlomo et al [24] found lower education was a predictor of parents' expectations to receive antibiotics for URTIs and in Trinidad, Mohan et al reported that general practitioners attributed antibiotic over-prescribing in general practice to parents' demands [25]. A proclivity to demand antibiotics was associated with decreased knowledge and in children from insured families higher rates of antibiotic use were associated with low antibiotic knowledge and a tendency to demand antibiotics [26]. In Hong Kong educated respondents and working guardians had higher knowledge scores, and those who knew the viral aetiology of URTIs were less likely to demand antibiotics [27]. In the present study, the rate of self-treatment with antibiotics by caregivers, was higher in those who had a high AKS (32%vs12%). This may be consequent to caregivers needing to report for work following quicker recovery of children whom they care for. Braun and Fowles found a correlation between the expectation to get antibiotic treatment and parents' occupation and parents who worked full time had higher expectations to get antibiotic treatment, assuming perhaps that antibiotics shorten disease duration and allow an earlier return to work [28]. In Trinidad and Tobago at least 25% of the population demand a prescription for antibiotics from a doctor and 21% keep antibiotics in the house for emergency purposes [19]. Educational campaigns for the public can correct the widespread misconceptions on antibiotic use and storage.

Earlier in describing the prescribing practices of Caribbean physicians we reported that respiratory tract infection was the most frequent reason for antibiotic prescriptions by physicians in the English and Dutch speaking Caribbean [29]. The influence of caregivers' (parents and relatives) knowledge on antibiotic use in children with URTIs has not been studied in any Caribbean region. Proportionately more children received antibiotics from caregivers for severe episodes of cold and cough, than for sore-throat and fever. Even for what they considered mild episodes (16%) of URTIs, caregivers administered antibiotics which as is current practice, probably obtained from community pharmacies on request [19]. In Malta parents gave antibiotics to their children without a prescription particularly for sore throat and the community pharmacy made the drugs available [30]. Caregivers in our study treated 44% of URTIs in children with antibiotics without consulting a physician or attending a health facility. We did not ascertain if the child suffered any unwanted effects of the drug, which information may have been important to discuss the consequences of freely giving antibiotics to children. We believe caregivers with health insurance, education beyond the primary level and a high AKS had obtained antibiotics informally at community pharmacies and those with a low knowledge score initiated medication with antibiotics from the home storage or given them by relatives and friends. A call for strict vigilance and enforced controls regarding 'over-the-counter' availability of antibiotics without a physician's prescription, despite being controlled drugs in Trinidad and Tobago, has been made in an earlier report [19].

Inappropriate antibiotic use is a common practice in the out patient setting in a clinic, or a physician's office [31,32], and has been attributed to the combination of time pressures of outpatient practice, diagnostic uncertainty, and physicians' misconceptions of patient expectations [33]. The cost and time spent for a visit to the health center or a physician's office and a genuine concern about the children's health could have pushed caregivers in Trinidad and Tobago to purchase antibiotics without a prescription which is a widespread practice here, and initiate treatment for common childhood respiratory tract illnesses. In Africa, Asia and Latin America antibiotics are obtained from pharmacies, hospitals and even from untrained vendors at the market place [21,34-36]. In Bavi, Vietnamese children were treated with antibiotics frequently by caregivers without physician consultation, resulting in a high prevalence of multi drug-resistant strains (MDR) among respiratory pathogens [37]. Contributing to the existence of the reservoir of MDR genes among bacterial pathogens undermines the effectiveness and success of antibiotic therapy in childhood respiratory tract infections in low-income countries.

## Conclusions

Inappropriate antibiotic use for paediatric URTIs in Trinidad and Tobago may have been facilitated by low knowledge, erroneous beliefs and easy availability of these drugs without the required prescription, at a retail pharmacy. A combination of education and communication to combat patients' expectations for treatment, and the physicians' appropriate prescription for antibiotics can halt inappropriate antibiotic use in children. Using narrow-spectrum antibiotics, promoting dialogue with caregivers to discuss symptom relief and antibiotic resistance, and encouraging active management of the child's illness with follow-up calls is recommended. Pharmacists have a serious responsibility not to dispense these agents without prescriptions and to discourage patients from obtaining these drugs for self-treatment. Widespread educational campaigns targeting the general public in Trinidad and Tobago, particularly parents and caregivers of young children should focus on the difference in bacterial and viral infections and the futility of treating viral infections with antibiotics. A multidisciplinary approach to rational antibiotic use, dispensing these drugs as 'prescription only medicine' and educating the public can halt inappropriate use and contain resistance.

## List of abbreviations

ABRS = Acute Bacterial Rhinosinusitis

AKS = Antibiotic Knowledge Score

SAHR = Sinus and Allergy Health Partnership

URTI = Upper Respiratory Tract Infection

## Competing interests

The author(s) declare that they have no competing interests.

## Authors'contributions

PP conceptualised the study and drafted the protocol, NP did data collection, and contributed to the draft manuscript, LMPP prepared the final manuscript. All authors contributed to the statistical analysis and the literature search.

## Pre-publication history

The pre-publication history for this paper can be accessed here:


